# Letter to the Editor: FIT Sensitivity—A Clinical Perspective

**DOI:** 10.3389/bjbs.2024.13444

**Published:** 2024-07-26

**Authors:** Eddie Cole, Deepa Narayanan, Ree Nee Tiam, John Shepherd, Mark O. R. Hajjawi

**Affiliations:** ^1^ York and Scarborough Teaching Hospitals NHS Foundation Trust, York, United Kingdom; ^2^ Hull University Teaching Hospitals NHS Trust, Hull, United Kingdom

**Keywords:** faecal immunochemical test (FIT), colorectal cancer (CRC), bowel cancer, sensitivity, haemoglobin

Dear Editors,

We would like to thank Dr. Jerjes for his interest in our paper [1] and very much welcome his positive feedback and personal perspective on our work. We would like to address some of the points raised in his letter and add our support to some of the national initiatives called for.

Our work focused on sensitivity and highlighted false negative FIT results. Our data showed most negative FIT results could be trusted to rule out colorectal cancer (CRC), but a minority of results were negative when cancer was present. We agree with Dr. Jerjes that this leaves the General Practitioner (GP) with a difficult diagnostic decision. When the GP is presented with a symptomatic patient who has a positive FIT, the patient still has a high probability of being cancer free [2, 3], but it is clear that the GP must refer urgently onto a 2 week wait cancer pathway for a definitive diagnosis. Therefore a “true” positive and a “false” positive FIT often lead to the same action by the GP. When presented with a negative FIT result in a symptomatic patient, the decision and action the GP must take is less clear. Therefore we welcome the roll out by NHS England of Rapid Diagnostic Centres, Community Diagnostic Centres, and non-specific symptoms cancer pathways which we believe are ideally suited to investigate FIT negative symptomatic patients using imaging techniques [4–6]. We would hope to see these centres develop into the multidisciplinary services called for, and that commissioners consider FIT in patient pathways.

In addition to the recommendations for primary and secondary care suggested by Dr Jerjes, we also suggest that providers review the health inequalities that may prevent a timely completion of a FIT by the patient. Sampling faeces using a FIT collection device is unpleasant and technically demanding for patients. When coupled with language barriers, learning disabilities and other physical ailments this task may become impossible.

As we stated in our paper, we support the clear recommendations from the Association of Coloproctology of Great Britain and Ireland (ACPGBI) and the British Society of Gastroenterology, and the recommendations of NICE DG56 that a negative FIT result should not prevent a colonoscopy [7, 8]. We are also keen to see the development of better diagnostic tools, including risk based algorithms [9] and molecular based tests [10, 11], but we note that further studies may still be required [12]. However, it must be noted that FIT and any future molecular tests are not diagnostic tests for CRC. They should be viewed as a tool to help decide who to perform the invasive diagnostic colonoscopy and biopsy on.

We agree that a holistic patient-centred-approach is best and that the patient should be aware of the significance of the FIT. This will empower them to be actively involved in making decisions about their own treatment. We strongly encourage GPs to “trust their gut feeling” when diagnostic tests do not match the clinical picture presented to them. We hope that our work can help guide service development across primary and secondary care and provide empirical evidence for any decisions made.

## Data Availability

The original contributions presented in the study are included in the article/Supplementary Material, further inquiries can be directed to the corresponding author.
